# A Rare Case of Nasal Sarcoma with BCOR Internal Tandem Duplication Showing Complete Pathologic Response to the VDC-IE Chemotherapy Protocol

**DOI:** 10.1155/2023/5546323

**Published:** 2023-12-26

**Authors:** Samer Salah, Maher A. Sughayer, Omar Jaber, Nebras Abu Abed, Fatena Ajlouni, Wisam Al Gargaz, Ramiz Abu Hijlih, Fawzi Abuhijla, Akram Al-Ibraheem, Farah Alul, Walid Naser

**Affiliations:** ^1^Department of Medical Oncology, King Hussein Cancer Center, Amman, Jordan; ^2^Department of Pathology, King Hussein Cancer Center, Amman, Jordan; ^3^Department of Radiology, King Hussein Cancer Center, Amman, Jordan; ^4^Department of Surgery, King Hussein Cancer Center, Amman, Jordan; ^5^Department of Special Surgery, Jordan University of Science and Technology, Irbid, Jordan; ^6^Department of Radiation Oncology, King Hussein Cancer Center, Amman, Jordan; ^7^Department of Nuclear Medicine, King Hussein Cancer Center, Amman, Jordan; ^8^Department of Cell Therapy and Applied Genomics, King Hussein Cancer Center, Amman, Jordan

## Abstract

Sarcoma with *BCOR* genetic alteration is an exceptionally rare and emerging subtype of sarcoma. It is categorized into two types: *BCOR*-related gene fusions such as *BCOR::CCNB3* sarcomas and other *BCOR*-rearranged sarcoma and sarcomas with internal tandem duplication of *BCOR* genes such as infantile undifferentiated round cell sarcomas and primitive myxoid mesenchymal tumors of infancy. *BCOR::CCNB3* sarcomas predominantly arise in bone rather than soft tissue and exhibit a higher occurrence in children and adolescent males, whereas sarcomas with *BCOR* internal tandem duplication show a wider age range but usually arise in the first year of life. Due to their rarity, there is ongoing debate and uncertainty regarding the best treatment approach, with a lack of specific clinical trials addressing these tumors. In this report, we present a unique case of sarcoma with internal tandem duplication of *BCOR* gene originating in the nasal region. The tumor was successfully and completely resected using the standard VDC-IE chemotherapy protocol, resulting in an unprecedented 100 percent tumor necrosis. The patient has completed the protocol and remains recurrence-free 13 months after diagnosis. This case suggests potential efficacy of the standard VDC-IE protocol in achieving remarkable responses in *BCOR* rearrangement sarcomas, including the internal tandem duplication subtype. However, further studies are needed to determine the optimal treatment strategies for this disease.

## 1. Introduction

Sarcoma with BCOR genetic alteration is an extremely rare form of sarcoma that shares morphological similarities with the Ewing sarcoma. Undifferentiated small round cell sarcomas usually exhibit relative morphological similarities to the Ewing sarcomas and were initially named as the Ewing-like sarcomas. Initially, they were divided into two main subtypes according to the detected gene fusions: CIC*::*DUX4 rearranged subtype and *BCOR::CCNB3* subtype. In contrast to CIC*::*DUX4 rearranged round cell sarcomas, *BCOR::CCNB3* rearranged sarcomas are more inclined to occur in the bone, whereas CIC rearrangement sarcomas tend to develop in soft tissues and to follow a more aggressive trajectory. Additionally, *BCOR::CCNB3* sarcomas display a higher occurrence in males, which is not observed in CIC-rearranged sarcomas [1–4].

It is important to mention that Pierron et al. were the pioneers in documenting this condition in 2012 through genome sequencing of cases of undifferentiated round cell sarcomas that did not exhibit any distinct genetic abnormalities [1]. In their publication, they identified a novel gene fusion, namely, *BCOR::CCNB3*. Since then, there has been a growing body of literature discussing this disease entity [2–5]. Nevertheless, most of the current evidence is based on case reports and series that include small number of patients [3, 4].

Sarcoma with *BCOR* genetic alteration encompasses different subtypes, with two notable ones being sarcoma with internal tandem duplication of BCOR gene (*BCOR-ITD*) and *BCOR*-related gene fusion such as *CCNB3*. *BCOR::CCNB3* fusion is a frequently observed genetic alteration in this category, characterized by the fusion of the *BCOR* and CCNB3 genes. This fusion event results in the formation of an abnormal protein product that plays a role in the development and progression of the sarcoma. On the other hand, the internal tandem duplication type refers to a type of *BCOR* rearrangement where the *BCOR* gene is duplicated, leading to an abnormal gene structure. Among these two subtypes, *BCOR::CCNB3* fusion is more commonly encountered in *BCOR* rearranged undifferentiated round cell sarcomas.

Due to the rarity of this sarcoma subtype, there is limited data on optimal treatment strategies or the optimal chemotherapy regimen. In fact, the use of the Ewing sarcoma protocols for managing these tumors has been debated [4]. It is important to note that no specific clinical trials have been conducted for *BCOR*-rearranged sarcomas.

In the current report, we present a unique case of a young male patient with nasal sarcoma with *BCOR* internal tandem duplication, who achieved successfully complete resection of the tumor with 100% pathologic tumor necrosis following the vincristine, doxorubicin, and cyclophosphamide (VDC) alternating with ifosfamide and etoposide (IE); which is the standard VDC-IE protocol utilized for the Ewing sarcoma.

## 2. Case Report

**Figure 1:**
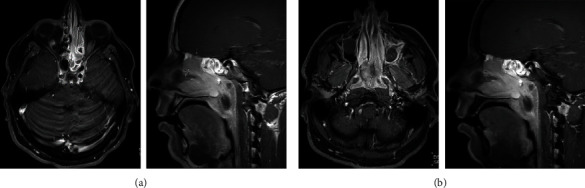
Contrast-enhanced T1 fat sat axial and sagittal MRI images showed two contrast-enhancing masses: one is nasoethmoidal (a), and the second one is in the nasal choana and postnasal space (b).

**Figure 2:**
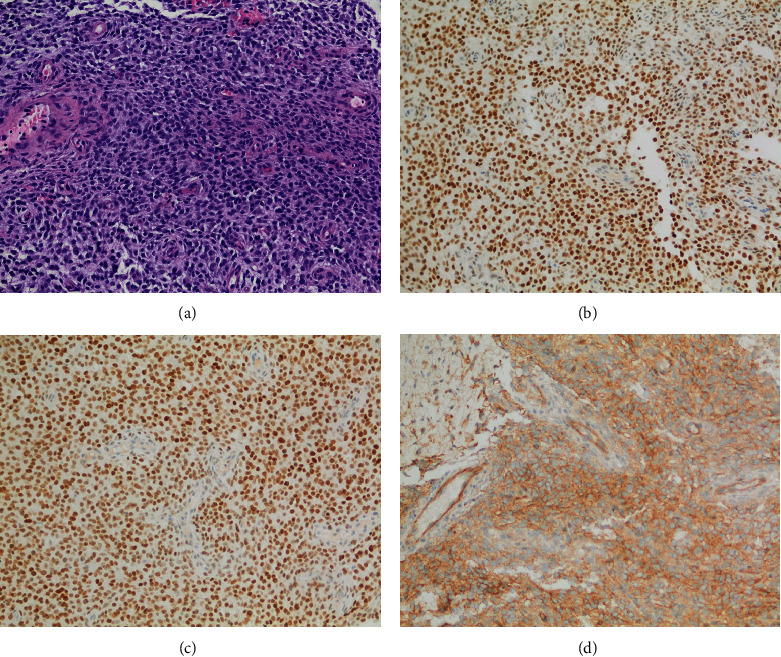
Sarcoma with *BCOR* internal tandem duplication. (a) The tumor is composed of atypical monomorphic cells with little pleomorphism. Hematoxylin and eosin, 200x. (b) The tumor cells are positive for BCOR immunohistochemistry, 200x. (c) The tumor cells are positive for SATB2 immunohistochemistry, 200x. (d) The tumor cells are focally positive for CD-99 immunohistochemistry. CD-99 immunohistochemical stain, 200x.

A 22-year-old male patient presented to our hospital in June 2022 with 4-month history of nasal obstruction, left-sided epistaxis, left-sided headache, and left earache. He underwent magnetic resonance imaging (MRI) of the sinuses outside our center, which showed a left nasal mass that seems to originate from the left maxillary sinus. He was then referred to our center following nasal biopsy which was reported outside as sarcoma, likely representing synovial sarcoma. MRI of head and neck in June 2022 showed two paranasal sinus lesions. The first appeared as a small localized contrast-enhancing left ethmoidonasal lesion, and the second one was a larger mixed-density nasochoanal mass extending to the postnasal space and upper nasopharynx (Figure 1). Computed tomography (CT) of the chest in June 2022 did not reveal lung metastasis. An FDG-PET scan on 19 July 2022 showed two moderately hypermetabolic malignant soft tissue nasal lesions, in keeping with the patient's known primary tumor. There was no convincing hypermetabolic potentially metastatic cervical lymphadenopathy as well as no any distant metastatic lesion. Pathology review of the nasal biopsy at our center showed a tumor composed of proliferation of atypical monotonous small round cells that were focally positive for CD99 and diffusely positive for BCOR, SATB2, and TLE-1 immunostains (Figure 2). The tumor cells were negative for SS18-SSX, NKX2.2, and STAT6, thus ruling out synovial sarcoma, Ewing's sarcoma, and solitary fibrous tumor, respectively. These findings were suggestive of undifferentiated round cell sarcoma with features of *BCOR*-altered sarcoma. Next-generation sequencing (NGS testing) of the tumor tissue specimen showed internal tandem duplication of *BCOR* gene, thus confirming the diagnosis.

**Figure 3:**
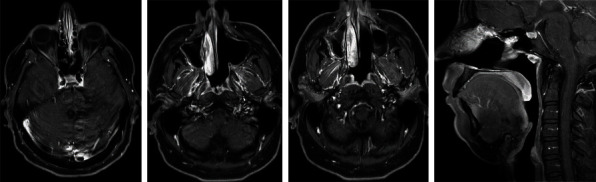
T1 post contrast fat sat axial (1st three) and sagittal (last one) MRI images showed complete resolution of the previous nasoethmoidal and nasochoanal/postnasal masses posttreatment (1st image is at the level of the ethmoid air cells, and rest of images are including the nasal choana and postnasal space).

The patient initiated chemotherapy with the standard VDC-IE Ewing sarcoma protocol on the 6th July 2022. Chemotherapy was initiated as alternating cycles of VDC and IE, every 3 weeks, for a total of 14 cycles. MRI neck on 20 Sep 2023, (following the 4th cycle) showed dramatic improvement, with resolution of the previously seen left nasoethmoidal and choanal/postnasal masses (Figure 3). CT chest continues to show no evidence of metastasis. He underwent surgical excision of the tumor on 6 Oct 2022. The pathology of the surgical specimen showed intranasal mucosa and bone trabeculae with necrosis and granulation tissue consistent with treatment-related changes and complete necrosis without any viable tumor seen. He completed chemotherapy on 21 May 2023. Last head and neck MRI and CT chest on 8 May 2023 showed no evidence of recurrence or metastasis. He tolerated chemotherapy very well and without appreciable toxicities.

## 3. Discussion

*BCOR*-rearranged round cell sarcomas are extremely rare tumors, with a more favorable outcome compared to CIC*::*DUX4 round cell sarcoma. We reviewed the literature and identified a total of 166 reported cases of *BCOR*-rearranged sarcoma [1–4, 6–17]. Molecular alterations included *BCOR::CCNB3* in 131 patients (79%), other *BCOR* fusions in 5 patients (3%), and *BCOR-ITD* in 30 patients (18%). Disease characteristics, treatment modalities, and outcomes of these patients are summarized (Table 1). A total of 127 (77%) of these cases were reported in males. Male predominance was more pronounced in papers reporting cases of *BCOR::CCNB3* as opposed to *BCOR-ITD* undifferentiated round cell sarcomas [4, 7, 11, 17]. Bone has almost been consistently reported as the most common origin [1, 2, 4, 10, 14]. Of note, most cases presented with localized disease [1, 2, 7, 11, 12].

Most patients with *BCOR* rearranged undifferentiated round cell sarcoma are treated with combined approach of surgery and chemotherapy [2, 4, 7, 13, 14]. However, there has been no consensus regarding the optimal regime owing to the lack of clinical trials. The use of the Ewing sarcoma regimens (VDC-IE, VIDE) has been commonly reported [1, 3, 4, 7, 8, 14, 15]. However, other utilized regimens included ifosfamide and doxorubicin [1, 2, 7] and osteosarcoma regimens such as platinum and anthracycline combinations and methotrexate [2, 4, 14]. A meta-analysis of 57 cases from 10 studies suggested that non-Ewing protocols are safe and potentially as effective as the Ewing protocols [5]. Nevertheless, the small number of patients in each of the studies, the retrospective design, and the lack of data on heterogeneity across studies included might be important limitations of that analysis.

While chemotherapy and surgery are commonly utilized, there is limited literature on postchemotherapy tumor necrosis. Pierron et al. reported that all 13 patients with *BCOR:CCNB3* with data on postchemotherapy necrosis had poor tumor necrosis [1]. Our case is unique compared to previous reports, as it represents the first report of complete pathologic tumor necrosis documented in the literature, suggesting the clinical effectiveness of the VDC-IE protocol for *BCOR*-rearranged round cell sarcomas. Additionally, the tumor's site of origin in our case is exceptionally rare, in contrast to the predominantly reported bone origin of this tumor [1, 2, 4, 10].

To conclude, we have shared an extraordinary case of sarcoma featuring *BCOR-ITD*, occurring in a rare site and demonstrating an exceptional response to the standard VDC-IE chemotherapy regimen. Notably, this is the first documented case in the literature to exhibit complete pathologic necrosis following the VDC-IE protocol. Due to its rarity, enrolling in clinical trials is difficult, highlighting the importance of multicenter collaboration to determine optimal systemic therapy for this disease.

## Figures and Tables

**Table 1:** Retrospective studies and case reports of patients with *BCOR*-rearranged undifferentiated round cell sarcomas.

Author	Number of cases	Gender/age	Molecular alteration; number of patients	Bone vs. ST	Localized vs. metastatic	Treatment	Necrosis/outcome
Tan et al. [6]	1	M: 21 years old	*BCOR::CCNB3*: 1	ST	Localized	Surgery and CTX	NR/NR
Suzuki et al. [3]	1	M: 12 years old	*BCOR::CCNB3*: 1	Bone	Localized	Surgery and CTX	NR/NED at 12 months
Pierron et al. [1]	26	M: 17. F: 9. Mean age: 13.6	*BCOR::CCNB3*: 26	Bone: 21; ST: 5	Localized: 24; metastatic: 2	Surgery with or without CTX: 12. CTX with or without surgery or RT: 24	Poor necrosis in 13. Not clear for others. AWD:3; NED: 16; DOD: 8
Puls et al. [4]	10	M: 9. F: 1. Mean age: 13.7	*BCOR::CCNB3*: 10	Bone: 7; ST: 3	Localized: 6; metastatic: 4	CTX+RT: 2. Surgery+CTX: 4. Surgery+CTX + RT: 4	NR/NED: 6; AWD: 1; DOD: 3
Peters et al. [7]	6	M: 6. Age: 7-13 years	*BCOR::CCNB3*: 6	Bone: 1; ST: 5	Localized: 6	Surgery: 2. Surgery+CTX: 1. Surgery+CTX + RT: 2. CTX + RT: 1	Not applicable. All CTX was adjuvant. NED: 4; DOD: 2
Shibayama et al. [8]	3	M: 3. Mean age: 13.6	*BCOR::CCNB3*: 3	Bone: 3	Localized: 3	Surgery+CTX: 3	NR/NED: 2; AWD: 1
Ludwig et al. [9]	11	M: 11. Mean age: 12.9	*BCOR::CCNB3*: 11	Bone: 6; ST: 5	NR	NR	NR/NED: 10; AWD: 1
Yamada et al. [10]	7	M: 2. F: 5. Mean age: 16.5	*BCOR::CCNB3*: 7	Bone: 6; ST: 1	NR	NR	NR
Matsuyama et al. [11]	11	M: 11. Mean age: 16.4	*BCOR::CCNB3*: 11	Bone: 4; ST: 7	Localized: 10; metastatic: 1	NR	NR/NED: 7; DOD: 1; NR: 3
Krskova et al. [12]	8	M: 6. F: 2. Mean age: 15.1	*BCOR::CCNB3*: 8	Bone: 3; ST: 5	Localized: 6; metastatic: 2	CTX + RT: some had surgery	NR/NED: 4; DOD: 3; AWD: 1
Kao et al. [2]	36	M: 31. F: 5. Mean age: 15.1	*BCOR::CCNB3*: 36	Bone: 20; ST: 14; kidney: 2	Localized: 32; metastatic: 4	Surgery+CTX + RT: 10. Surgery+CTX: 8. Surgery alone: 8. CTX and RT: 1. No data: 9	NR/NED: 13; DOD: 2; DUC: 1; AWD: 6; NR: 14
Rekhi et al. [13]	5	M: 4. F: 1. Mean age: 26	*BCOR::CCNB3*: 5	Bone: 2; ST: 3	NR	CTX+surgery: 2. CTX + surgery+RT: 1. No data: 2	NR/AWD: 2; DOD: 1; others: NR
Brady et al. [14]	5	M: 4. F: 1. Mean age: 14	*BCOR::CCNB3*: 5	Bone: 5	NR	Surgery+CTX: 5	NR/NED: 3; DOD: 1; AWD and ongoing treatment: 1
Malik et al. [15]	1	M: 13 years old	*BCOR-ITD*	Bone	Localized	Surgery+CTX	50% necrosis. Outcome was NR
Yang et al. [16]	2	M: 17 years old. F: 7 months	*BCOR-ITD*: 1, *BCOR::CCNB3*: 1.	Bone: 1; ST: one	NR	NR	NR/NR
Antonescu et al. [17]	33	M: 19. F: 14. Age: 19 patients < 1 year. Two were diagnosed at age 1-2 years, other two at 16 and 17 years	*BCOR ITD*: 28. *BCOR- YWHAE-NUTM2B/E* fusion: 4. *BCOR- YWHAE* fusion: 1	Bone: 5; ST: 28	Not clear	Data on 23 patients: surgery+CTX: 14. Surgery +CTX + RT: 4. CTX alone: 5	Necrosis reported for 2 patients: one had >50% necrosis, and the other had 0% necrosis. Outcome data available for 25 patients: DOD: 14; died of other cause: 1; NED: 6; AWD: 4

M: male; F: female; ST: soft tissue; CTX: chemotherapy; RT: radiotherapy; NR: not reported; NED: no evidence of disease; AWD: alive with disease; DOD: died of disease.

## Data Availability

The pathology and radiology data used to support the findings of this study are included within the article. Other data (e.g., other pathology and radiology data) are available from the corresponding author upon request.
